# ER Stress and Unfolded Protein Response in Cancer Cachexia

**DOI:** 10.3390/cancers11121929

**Published:** 2019-12-03

**Authors:** Anirban Roy, Ashok Kumar

**Affiliations:** Department of Pharmacological and Pharmaceutical Sciences, College of Pharmacy, University of Houston, Houston, TX 77204, USA; aroy24@central.uh.edu

**Keywords:** Skeletal muscle, cancer cachexia, ER stress, PERK, IRE1, ATF4, XBP1, ATF6, signaling

## Abstract

Cancer cachexia is a devastating syndrome characterized by unintentional weight loss attributed to extensive skeletal muscle wasting. The pathogenesis of cachexia is multifactorial because of complex interactions of tumor and host factors. The irreversible wasting syndrome has been ascribed to systemic inflammation, insulin resistance, dysfunctional mitochondria, oxidative stress, and heightened activation of ubiquitin-proteasome system and macroautophagy. Accumulating evidence suggests that deviant regulation of an array of signaling pathways engenders cancer cachexia where the human body is sustained in an incessant self-consuming catabolic state. Recent studies have further suggested that several components of endoplasmic reticulum (ER) stress-induced unfolded protein response (UPR) are activated in skeletal muscle of animal models and muscle biopsies of cachectic cancer patients. However, the exact role of ER stress and the individual arms of the UPR in the regulation of skeletal muscle mass in various catabolic states including cancer has just begun to be elucidated. This review provides a succinct overview of emerging roles of ER stress and the UPR in cancer-induced skeletal muscle wasting.

## 1. Introduction

Cachexia is a multifactorial syndrome characterized by progressive loss of skeletal muscle mass and functional impairment. The condition primarily contributes to higher rates of morbidity and mortality in terminal illnesses such as chronic obstructive pulmonary disease, chronic kidney disease, rheumatoid arthritis, and cancer [1]. The major symptoms of cachexia include anorexia, inflammation, pronounced muscle wasting which often involves wearying of chest muscles, diaphragm, and heart leading to respiratory or cardiac failure in severe cases. During the preceding stages of cachexia, patients tend to lose more than 5% of their total body weight which primarily involves skeletal muscle mass and occasionally accompanies deletion of fat mass [2]. Moreover, cachexia is irreversible and supplementation with conventional nutritional support fails to prevent the condition thereby acting as a hindrance to palliative care, patient management, and quality-of-life in affected individuals [3,4]. Almost 50–80% of cancer patients suffer from cachexia where the complications are further escalated accounting for nearly 20% of deaths directly related to cachexia [1,5]. Cancer-associated cachexia also diminishes the therapeutic efficacy of anti-cancer treatments and decreases survival rate which is believed to be a consequence of cachexia-induced systemic inflammation [6]. Additionally, cachectic individuals are more likely to become resistant to cancer therapy [7,8]. Moreover, as chemotherapeutic drugs are dosed on the basis of surface area, excessive loss of skeletal muscle mass and fat can change a chemotherapeutic drug’s volume of distribution causing intensification and susceptibility of chemotherapy related side-effects [9]. Recent studies demonstrating that cancer-induced muscle wasting fuels tumor progression and prevention of muscle wasting prolongs the survival of tumor-bearing hosts [10,11] further emphasize the urgency of elucidating the mechanisms of muscle wasting to facilitate the development of new therapeutic approaches for cancer patients.

**Figure 1:**
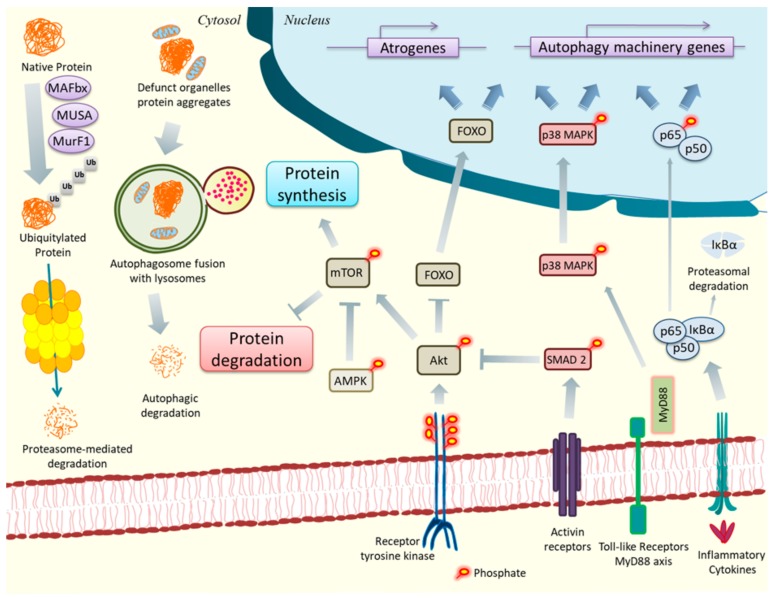
Major pathways involved in muscle loss in cancer cachexia. The ubiquitin-proteasome system and autophagy are the major modes of proteolysis in cancer cachexia. Native proteins destined to non-lysosomal degradation are polyubiquitylated by E3 ubiquitin ligases like MuRF1, MAFbx, and MUSA before being fragmented into smaller peptides by the 26S proteasome. Autophagy removes defunct organelles and protein aggregates by assembling them into phagophores followed by autophagosome formation. Autolysosomes are then formed by fusion of autophagosomes with lysosomes, which degrade the engulfed components. Growth stimulating ligand binds to receptor tyrosine kinases to phosphorylate and activate Akt, which consequently triggers mammalian target of rapamycin (mTOR) pathway to increase rate of protein synthesis and decrease protein degradation. SMAD2 and the energy sensor kinase AMPK can also regulate cellular protein level by inhibiting Akt and mTOR, respectively. Phosphorylated Akt also prevent the nuclear localization of FOXO proteins by phosphorylating them, however, in cachexia, pAkt is inhibited resulting in mTOR pathway suppression and nuclear translocation of FOXO proteins. FOXO proteins in nucleus trigger transcription of genes encoding proteins for autophagy mechanism and atrogenes like MAFbx, MuRF1, and MUSA. Transcription of these genes are also promoted by NF-κB and p38 MAPK activated by pro-inflammatory cytokines, Toll-like receptor/MyD88, Activin/Activin receptor axis.

Skeletal muscle wasting is attributed to heightened activity of ubiquitin-proteasome system (UPS) that causes intracellular protein degradation in a wide variety of catabolic conditions [12,13]. Muscle atrophy F-box (MAFbx/Atrogin-1), Muscle RING-finger protein-1 (MuRF1/TRIM63), neural precursor cell-expressed developmentally downregulated gene 4.1 (Nedd4.1), TNF-associated factor 6 (TRAF6), muscle ubiquitin ligase of SCF complex in atrophy-1 (MUSA), and recently discovered Specific of Muscle Atrophy and Regulated by Transcription (SMART) are specific E3 ubiquitin ligases that have been found to be persistently up-regulated in different models of cancer cachexia [14,15,16]. While the ubiquitin-proteasome system degrades most soluble proteins, eukaryotic cells also employ autophagy as a quality control mechanism to remove dysfunctional or damaged organelles, as well as aberrantly folded proteins [13,15]. Furthermore, under nutrient limiting conditions, stimulation of autophagy disintegrates organelles, long-lived soluble proteins, and protein aggregates into smaller biomolecules to feed the biosynthetic pathways that maintain muscle structure and function (Figure 1). However, catabolic conditions such as cancer cachexia, fasting, or disuse state triggers a prolonged and exuberant increase in autophagy flux resulting in extensive muscle wasting [12,17,18]. In addition to UPS and autophagy, other effector proteases, such as calpains and caspases can also independently degrade muscle proteins in some conditions [19,20]. The gene expression and the activity of various proteolytic systems is regulated through the activation of p38 MAPK, phosphatidylinositol-3-kinase (PI3K)/Akt/ mammalian target of rapamycin (mTOR), and nuclear factor-kappa B (NF-κB) signaling pathways [21]. Moreover, the PI3K/Akt/mTOR is a major pathway that increases the rate of protein synthesis leading to muscle hypertrophy (Figure 1). This pathway also inhibits protein degradation through suppressing the UPS and autophagy in skeletal muscle in catabolic states [21,22].

Cancer cachexia is often considered to be an energy wasting syndrome where increased catabolic processes causes an overall increase in resting energy expenditure leading to muscle atrophy and wasting. Hypermetabolism observed in many experimental models of cancer cachexia has been attributed to inefficient ATP production due to mitochondrial dysfunction and abnormalities, and aberrant electron transport chain activity, as well as altered mitochondrial membrane fluidity [23,24]. Dysfunctional mitochondria generate exuberant levels of reactive oxygen species causing oxidative damage to lipids and proteins rendering them nonfunctional. This deregulated redox homeostasis is a hallmark of cancer and increased reactive oxygen or nitrogen species (ROS/RNS) correlates with enhanced protein breakdown and inflammation [25,26,27]. Other reports suggest that increased activity of skeletal muscle mitochondrial uncoupling proteins (UCPs), UCP1, and UCP2, in models of cachectic tumors [28,29,30] disrupts the proton electrochemical gradient across the mitochondrial membrane leading to uncoupling of oxidative phosphorylation and production of heat instead of efficient ATP production.

The hyperactive protein metabolism in cancer patients has been speculated to be the result of altered expression patterns of pro-inflammatory mediators and cytokines [31]. Moreover, cachectic patients have increased activin levels in circulation. Activin being a negative regulator of muscle mass has been suggested to play a dominant role in the development of cachexia, although further studies are necessary to understand the underlying mechanism [32]. Additionally, tumor cell-derived extracellular vesicles can also aggravate muscle wasting. Recently, it was reported that heat shock proteins, Hsp70 and Hsp90 are packed and subsequently released in vesicles from tumor cells. These proteins bind to toll-like receptor (TLR) 4 on muscle cells leading to the activation of catabolic signaling pathways and production of inflammatory cytokines, IL-6 and TNF-α. in circulation [33]. Consistent with increased signaling from TLRs, we recently reported that levels of several TLRs and myeloid differentiation primary response gene 88 (MyD88), an essential component of TLR-mediated activation of downstream signaling, are increased in skeletal muscle of Lewis lung carcinoma (LLC) tumor-bearing mice [34]. Interestingly, addition of conditioned media of LLC cells or C26 adenocarcinoma induces the gene expression of several TLRs and MyD88 in cultured myotubes suggesting that factors produced by cachectic tumor cells upregulate the TLR-mediated signaling in skeletal muscle. Importantly, targeted ablation of MyD88 prevented LLC tumor-induced muscle wasting in mice. Ablation of MyD88 also prevented the activation of p38 MAPK and NF-κB pathways and gene expression of a few components of UPS and autophagy in skeletal muscle of LLC tumor-bearing mice [34]. Consistent with our findings with LLC tumor-bearing mice, another study recently demonstrated that genetic ablation of MyD88 attenuates muscle wasting and improves survival in a mouse model of pancreatic cancer cachexia further suggesting that the TLR/MyD88 signaling axis mediates the loss of skeletal muscle mass during cancer cachexia [35].

## 2. ER Stress, UPR, and Cancer Cachexia

The ER in eukaryotes forms a cell-type specific framework consisting of branching tubules and flattened membrane-enclosed sacs known as cisternae. It is the primary site for protein synthesis and folding, lipid synthesis, detoxification of chemical compounds, glucose metabolism, and calcium storage [36]. Moreover, the chaperones, foldases, and posttranslational modification proteins present in ER mediate proper folding of nascent transmembrane and secretory proteins into their native conformation followed by their assembly. ER also has a quality control mechanism for exporting only properly folded protein whereas terminally misfolded proteins are degraded by a process called ER-associated degradation (ERAD) [37,38]. However, the ER homeostasis sustaining vital cellular functions is often disturbed in disease conditions or due to changes in the microenvironment, temperature and reactive oxygen species, or by chemical compounds which results in heightened stress in the ER. To restore homeostasis and improve survival, cells initiate a signaling cascade commonly referred to as the unfolded protein response (UPR) [39]. The UPR is controlled by three transmembrane proteins, namely protein kinase R (PKR)-like endoplasmic reticulum kinase (PERK), inositol-requiring protein 1α (IRE1α), and activating transcription factor 6 (ATF6) that are activated to alleviate ER stress. In the absence of stress, intra-luminal domains of PERK, IRE1, and ATF6 bind to the ER luminal protein glucose-regulated protein 78 (GRP78), also known as heat shock protein A or binding immunoglobulin protein (BiP). However, accumulation of misfolded and/or unfolded proteins in the ER lumen leads to the dissociation of PERK, IRE1, and ATF6 from GRP78 thereby activating the downstream signaling cascades. The UPR activation is regulated by GRP78 due to its higher binding affinity towards unfolded or misfolded proteins compared to intra-luminal domains of PERK, IRE1, and ATF6 [40,41]. While the main function of the UPR is to restore homeostasis and promote cell survival, unmitigated ER stress leads to inflammation, metabolic abnormalities, and cell death in many tissues [42]. Emerging evidence further suggest that each arm of the UPR may have a distinct role in the regulation of cell fate in naïve and diseased states [42,43,44].

ER stress and disruptions in the UPR, calcium homeostasis, and proteostasis have been reported in clinical and experimental cachexia and other inflammation-driven muscle diseases such as myositis, suggesting a link between increased levels of inflammatory cytokines and ER stress in skeletal muscle [45]. Recent studies have further demonstrated that the markers of ER stress and the UPR are elevated in skeletal muscle in various catabolic conditions, including cancer [46,47,48]. TRAF6 is an E3 ubiquitin ligase and an adaptor protein that is involved in the downstream activation of multiple signaling pathways in response to cytokines and microbial products. Genetic ablation of TRAF6 attenuates skeletal muscle wasting in response to starvation, denervation, and tumor growth potentially through inhibition inflammatory signaling pathways and proteolytic systems [49,50]. Interestingly, TRAF6 is also involved in the activation of the UPR because targeted ablation of TRAF6 reduces the levels of ATF4, CHOP, GRP94, and GADD34 in skeletal muscle of fasted mice [49]. These findings suggest that activation of the UPR may involve signaling cross-talk with other pathways involved in the regulation of skeletal muscle mass. Further evidence about a link between ER stress and cancer induced skeletal muscle wasting was observed in LLC and APC^Min/+^ models of cancer cachexia [51]. In tumor bearing mice, the ER stress markers such as p-eIF2α, CHOP, IRE1α, XBP1, ATF6, DR5, GRP78, GRP94, GADD34, and sXBP1 were found to be substantially elevated as well as correlated with increased ubiquitylated protein levels and heightened expression of E3 ligases: MAFBx, MuRF1, TRAF6. The activation of ER stress during cancer cachexia is attributed to the factors produced by tumor cells. Indeed, treatment of cultured myotubes with conditioned medium from LLC cells increases the phosphorylation of eIF2α, alternative splicing of XBP1, and gene expression of several ER stress markers [51]. A recent study by Barreiro et al. indicates elevated marker of all the three arms of the UPR in vastus lateralis muscle in lung cancer cachectic patients. However, a caveat of the study was that while non cachectic lung cancer patients should have been used as a control, the comparisons were made with healthy subjects [48]. Intriguingly, instead of providing protection, pharmacological inhibition of ER stress using 4-phenylbutyrate (4-PBA), a chemical chaperone that attenuates ER stress [52,53], was found to exacerbate the loss of muscle mass in LLC tumor-bearing mice. Moreover, chronic administration of 4-PBA leads to significant loss of skeletal muscle mass and strength in naïve mice. Similarly, 4-PBA induces rapid atrophy in cultured myotubes, which is further increased in the presence of LLC conditioned medium. The study also provided evidence that 4-PBA inhibits the rate of protein synthesis and Akt/mTOR pathway and augments the activation of proteolytic systems during cancer cachexia [51] suggesting that some level of ER stress may be essential for maintaining skeletal muscle mass and health even in naïve conditions. While the markers of ER stress and the UPR are increased in skeletal muscle of LLC tumor-bearing mice and APC^Min/+^ mice, a recent study suggested that a few markers of ER stress are reduced in skeletal muscle of C-26 adenocarcinoma-bearing mice. Interestingly, blocking activin receptor type 2 (ACVR2) ligands using soluble activin receptor 2B fused to the Fc domain (sACVR2B-Fc) improved muscle mass and restored the UPR in skeletal muscle [54]. These findings further suggest that the UPR may have distinct role in the regulation of muscle mass in various models of cancer cachexia.

However, as ER stress response effectuates through the independent activation of IRE1, PERK, and ATF6 pathways along with added signaling regulation and crosstalk, it is imperative to understand the role of individual arm of the UPR in distinct models of cancer cachexia.

## 3. PERK and Cachexia

PERK is ubiquitously expressed type-I transmembrane serine threonine kinase with an ER luminal domain attached to GRP78 and a cytoplasmic kinase domain. PERK is involved in the proliferation, differentiation, and survival of different cells types [42,46]. *Perk1*-null mice die during the post-natal period due to β-cell degeneration the pancreas resulting in diabetes mellitus, reduced secretion of digestive enzymes, and defects in skeletal formation [38,55,56,57]. Similarly, homozygous *Eif2α*S51A mutant mice at perinatal stage due to metabolic defects leading to severe hypoglycemia [58]. Accumulating misfolded proteins in ER lumen results in GRP78 dissociation from PERK followed by oligomerization, trans-autophosphorylation, and activation. Like PKR, activated PERK phosphorylates nuclear factor (erythroid-derived)-like 2 (Nrf2) in response to ER stress leading to the release of Nrf2 from its inhibitor Keap1 and subsequent nuclear translocation to drive transcription of antioxidant response genes [59,60]. Additionally, activated PERK phosphorylates the serine 51 residue of eukaryotic initiation factor 2α (eIF2α). Phosphorylated eIF2α inhibits the eukaryotic translation initiation factor 2B (eIF2B) to suppress the rate of protein translation and folding. Interestingly, phosphorylated eIF2α selectively up-regulates the translation of ATF4 transcripts which is normally inefficiently translated. ATF4 up-regulates the expression of adaptive genes involved in protein homeostasis, resisting oxidative stress, and autophagy response [61]. Moreover, ATF4 and p-eIF2α can increase the translation of other stress responsive genes such as C/EBP homologous protein (CHOP), and BiP [62]. Furthermore, CHOP, ATF4, and p-eIF2α of the PERK signaling pathway are involved in terminating UPR and restoring the normal cellular functioning after ER stress has been relieved by positively up-regulating the expression of growth arrest and DNA-damage-inducible 34 (GADD34) protein. GADD34 acts as a negative feedback regulator of the UPR pathway by engaging with the catalytic subunit of type 1 protein serine/threonine phosphatase (PP1) to dephosphorylate p-IF2α and consequently causing de-escalation of UPR response and restoration of protein translation [61]. However, during persisting ER stress, ATF4 and CHOP stimulate the gene expression of molecules involved in cell cycle arrest, cell death, and delays feedback inhibition of p-IF2α by GADD34 [63,64].

**Figure 2:**
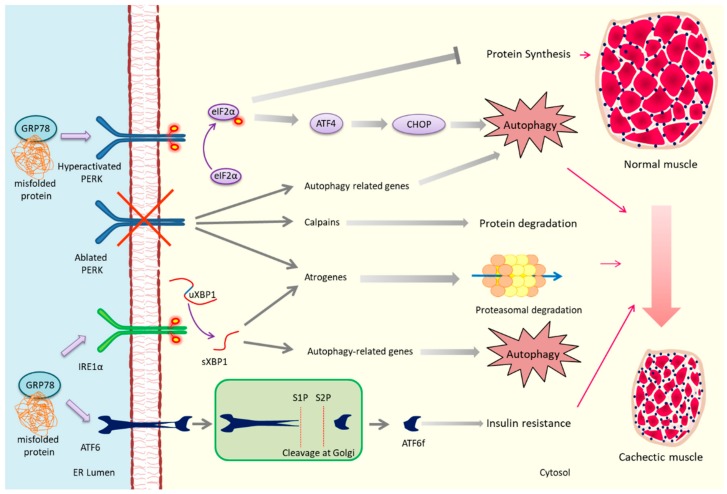
Putative mechanisms of action of unfolded protein response (UPR) in cancer cachexia. Misfolded proteins accumulated in the endoplasmic reticulum (ER) lumen bind to glucose-regulated protein 78 (GRP78) resulting in its dissociation from intraluminal domains of ER stress sensors protein kinase R-like endoplasmic reticulum kinase (PERK), IRE1α, and activating transcription factor 6 (ATF6) to consequently trigger their activation. PERK undergoes dimerization and trans-autophosphorylation before phosphorylating and activating eIF2α. Under conditions when PERK is hyper-activated, p-eIF2α inhibits protein synthesis and activates activating transcription factor 4 (ATF4). ATF4 up-regulates C/EBP homologous protein (CHOP) expression which is responsible for inducing autophagy machinery genes. Complete deletion of PERK gene also induces the expression of atrogenes, autophagy promoting genes and calpains. The endonuclease domain of activated IRE1α splices the unspliced XBP1 (uXBP1) to spliced form (sXBP1). sXBP1 escalates the gene expression of atrogenes and autophagy-related molecules to effectuate cachexia. The ER sensor ATF6 is translocated to Golgi following its activation where it is sequentially cleaved by site1 protease (S1P) and site2 protease (S2P) proteases. The cleaved fragment ATF6f promotes cachexia by mediating insulin resistance in skeletal muscle.

The PERK arm of UPR is involved in many aspects of skeletal muscle physiology and pathophysiology. For example, the components of PERK/eIF2α regulate survival, proliferation, and differentiation of myoblasts during myogenesis [46]. Moreover, PERK also regulates the self-renewal, survival, and myogenic potential of satellite cells during regenerative myogenesis [65,66]. Contrarily to its pro-survival role in muscle progenitor cells, inducible activation of PERK can also contribute to muscle wasting [67,68]. Recent studies using skeletal muscle-specific Fv2E-PERK transgenic (Tg) mice have demonstrated that inducible activation of PERK leads to skeletal muscle wasting in adult mice in a dose-dependent manner [67,68]. Interestingly, inducible activation of PERK also leads to increased gene expression of several enzymes involved in amino acid metabolism and upregulation of antioxidant molecule, glutathione in skeletal muscle tissues [68]. The study also showed that spurious activation of PERK increases the gene expression and secretion of fibroblast growth factor 21 (FGF21), an anti-obesity factor which stimulates energy expenditure in brown adipose tissue [68]. These findings suggest that in addition to causing atrophy, hyper-activation of PERK improves metabolic adaptation of the whole body (Figure 2).

Levels of a few ER stress markers, including CHOP, are increased in skeletal muscle upon denervation. Elevated levels of ER stress markers have also been observed in skeletal muscle of AR113Q mice, a model of spinal and bulbar muscular atrophy [69]. Surprisingly, genetic ablation of CHOP exacerbates denervation-induced muscle wasting in mice attributed to the stimulation of autophagy. Inhibition of autophagy attenuates denervation-induced muscle atrophy and increases the lifespan of AR113Q mice [69]. Nevertheless, recent studies in our laboratory suggested that some level of PERK activation is required for maintaining skeletal muscle mass and tonicity. Targeted ablation of PERK leads to muscle wasting and decline in contractile function [70]. Skeletal muscle of PERK-knockout mice show mild atrophy and substantially reduced force production during isometric contractions. Similarly, shRNA-mediated knockdown or pharmacological inhibition of PERK produced atrophic characteristics in cultured myotubes. While the exact mechanisms remain unknown, genetic ablation of PERK resulted in increased gene expression of components of UPS and autophagy, heightened activation of calpains, disrupted protein turnover, and aberrant regulation of FGF19 subfamily members in skeletal muscle [70].

The markers of the PERK arm of the UPR are increased in skeletal muscle of LLC tumor-bearing mice [51]. Intriguingly, loss of muscle mass was more pronounced in LLC tumor-bearing PERK-knockout mice suggesting an important role of PERK in maintaining muscle mass and contractile function during cancer cachexia [70]. Previous studies have shown that ATF4, which is also activated by PERK/eIF2α arm of the UPR, is involved in skeletal muscle atrophy during starvation [49,71]. Levels of ATF4 are increased in skeletal muscle of LLC tumor-bearing cachectic mice and its expression was inhibited when ER stress was attenuated using 4-PBA or through genetic ablation of PERK [51,70]. However, under fasting conditions, ATF4 can also stimulate the expression of growth suppressor genes p21 (Cip1/Waf1, GADD45α, PW1/Peg3, and CARP [71,72]. Thus, whether ATF4 expression induced growth suppression results in cachexia or is a defense mechanism to prevent further nutrient utilization remains under speculation.

## 4. IRE1/XBP1 and Cachexia

The ubiquitously expressed IRE1α is encoded by the endoplasmic reticulum to nucleus signaling 1 (ERN1) gene in humans [73] and is involved in regulated protein folding capacity and cell fate determination [74]. IRE1α has a conserved and structurally homologous region with PERK which is necessary and sufficient for dimerization and UPR signaling [75]. Similarly to PERK, dissociation of GRP78 triggers the sequential oligomerization of IRE1α, activation of its cytosolic kinase domain and trans-autophosphorylation at Ser724, Ser726, and Ser729 residues. However, phosphorylated IRE1α acts as an endonuclease and in a spliceosome independent manner catalyzes the splicing of human XBP1 mRNA into spliced isoform of XBP1 (sXBP1) along with a 26 nucleotide intron. Although both unspliced and spliced forms of XBP1 are translated, only sXBP1 acts as a transcription factor due to the presence of a transactivation domain. sXBP1 is a basic leucine zipper transcription factor and promotes the transcription of chaperones, foldases, and many components of the ER-associated degradation (ERAD) in response to ER stress [42,43,74]. However, with persistent ER stress, increased IRE1α activity also activates c-Jun N terminal kinase (JNK) and nuclear factor-kappa B (NF-κB) pathways [43]. The physiological significance of IRE1/XBP1 axis is highlighted by the findings that *Ire1^−/−^* and *Xbp1*^−/−^ mice die embryonically due to liver failure, defects in the placenta, and anemia [76,77].

In myogenic cells, the expression of XBP1 is governed by MyoD and myogenin where it regulates the expression of genes involved in ER function, cell growth, and DNA repair pathways [78,79]. In addition to misfolded protein accumulation, the IRE1α-XBP1 axis of the UPR can also be activated by agonists of Toll-like receptors (TLRs) or by fluctuating glucose levels and subsequently function to regulate innate immunity and maintain insulin levels, respectively [80,81]. We have recently reported that TLRs regulate the activation of PERK and IRE1α arms of the UPR in skeletal muscle of LLC tumor-bearing mice [34]. Interestingly, targeted ablation of XBP1 attenuates the muscle wasting of LLC tumor-bearing mice. Further, the activation of NF-κB and p38 MAPK signaling and gene expression of several components of UPS and autophagy were reduced in skeletal muscle of XBP1-knockout mice compared to controls in response to LLC tumor growth [34]. Moreover, shRNA-mediated knockdown of sXBP1 prevents LLC− or C26 cells conditioned media-induced atrophy in cultured myotubes. Consistent with a role of XBP1 in muscle wasting, overexpression of sXBP1 using an adenoviral vector reduces myotube diameter with heightened expression of markers for the UPS and autophagy. Altogether, our study provided initial evidence that the activation of ER stress sensor sXBP1 mediates skeletal muscle wasting in normal as well as in cancer conditions [34]. It is possible that ER stress or signaling from TLRs stimulates IRE1α/sXBP1 signaling axis to mediate muscle wasting as the activity of sXBP1 is dependent upon IRE1α. However, ER stress-induced IRE1α activation can also regulate cellular growth and apoptosis by involving MAPK pathways where the endonuclease activity of IRE1α is dispensable. It has been reported that overexpression of IRE1α results in oligomerization and activation of IRE1α kinase domain which interacts with TRAF2 to trigger c-Jun N-terminal kinase (JNK) activation and initiate a pro-apoptotic response independent of sXBP1 activation. Additionally, the pro-apoptotic proteins Bax, Bcl-2, PUMA, and BIM can also interact with the cytosolic domains of IRE1α resulting in its activation [59]. Interestingly, IRE1α is also involved in ER stress-induced ERK1/2 activation and thereby promoting cell survival under certain conditions. Therefore, the implication of selective IRE1α activation in absence of ER stress has to be investigated to reveal its role in cancer cachexia (Figure 2).

## 5. ATF6 and Cachexia

The human ATF6 transcription factor is constitutively synthesized as a type II transmembrane precursor protein and is encoded by ATF6α and ATF6β genes. While genetic deletion of ATF6α or ATF6β does not cause any developmental defects, double deficiency of ATF6α and ATF6β is embryonically lethal suggesting a critical role of ATF6α/β in development [82]. Moreover, ATF6α^-/-^ produce lethality when challenged with ER stress agents, possibly due to liver dysfunction [83]. ER stress mediated activation of ATF6 is followed by its export to the Golgi where it is sequentially cleaved by Golgi-membrane bound proteases; site1 protease (S1P) and site2 protease (S2P) into a ~400 residue long cytosolic N-terminal fragment ATF6f. ATF6 contains a transcriptional activation domain, a bZIP domain, and a DNA-binding domain, as well as nuclear localization signals which enable its nuclear export [82,84]. It interacts with other bZIP containing proteins and transcription factors such as CREB, CREB3L3, and XBP1 to induce gene expression of UPR molecules involved in alleviating ER stress [85,86].

ATF6 has been found to play an important role in skeletal muscle development, post-exercise recovery, and glucose metabolism. ER stress signaling mediated specifically by the ATF6 arm of UPR has been reported to trigger caspase-dependent apoptosis in subsets of myoblasts susceptible to cellular stress suggesting that ATF6 transmits ER stress signal leading to developmental apoptosis during myotube formation [87]. Moreover, reports suggest that ATF6 also enables efficient recovery of skeletal muscle after acute exercise by interacting with peroxisome proliferator-activated receptor gamma coactivator-1 alpha (PGC-1α) [88]. PGC-1α is a key transcription factor that is involved in mitochondrial biogenesis as well as function; and PGC-1α overexpression imparts better performance capability and adaptability to exercise in transgenic mice [14]. Additionally, ATF6 plays a regulatory role in glucose metabolism and transport in skeletal muscle. Glucose is imported in skeletal muscle by GLUT4 transporter and GLUT4 expression is regulated by MEF2A. Expression of GLUT4 is dependent on the transcriptional activity of MEF2A and its co-activator, PGC-1α. A recent report demonstrates that glucosamine, high glucose, and ER stressor, Thapsigargin inhibit the expression of GLUT4, MEF2A, and PGC-1α by activating the gene expression of ATF6, phosphorylation of GRP78, and sXBP1 levels in rat and human skeletal muscle cells. Furthermore, the down-regulation of GLUT4, MEF2A, and PGC-1α were also achieved by overexpression of ATF6 and silencing ATF6 completely prevented glucosamine or Thapsigargin-mediated inhibition of GLUT4, MEF2A, and PGC-1α gene expression suggesting the role of ATF6 arm of the UPR in insulin resistance [89]. Even though insulin resistance precedes muscle wasting, it remains enigmatic whether ATF6-mediated insulin resistance fosters cachectic phenotype. During physiological conditions, insulin binding to its receptor which results in the activation of the PI3K/Akt signaling pathway that consequently inhibits FOXO transcription factors, caspase-3, and downregulates the expression of E3 ubiquitin ligases atrogin-1 and MuRF-1. During cancer cachexia, which is a state of insulin resistance, the activity of PI3K is decreased relieving FOXO and caspase 3 inhibition and activation of ATP-dependent ubiquitin-proteasome pathway [89]. Thus, whether ATF6 mediated insulin resistance promotes cachectic conditions during cancer remains to be investigated, although ATF6 levels have been found to remain elevated in mice bearing LLC tumor as well as in APC^Min/+^ cancer models of cachexia [51].

## 6. Concluding Remarks

From the above discussion, it is evident that the ER stress-induced UPR pathways regulate myogenic cell survival, self-renewal, differentiation, and dedifferentiation in various physiological and pathophysiological states. It is also now increasing clear that individual arms of the UPR may have distinct effect on the regulation of skeletal muscle mass and function. Although recent studies in genetically modified mouse models and gene manipulation techniques in cultured muscle cells suggest the involvement of ER stress and the UPR in cancer cachexia yet the intracellular conditions dictating the UPR to act as a pro-cachectic or anti-cachectic signal is still unknown. For instance, it has been speculated that PERK hyper-activation mediates cachectic phenotype; yet a basal level of PERK activation is essential to prevent the deleterious wasting effects in skeletal muscle. Likewise, ATF6 pathway which is known to be involved in initiating an adaptive response upon ER stress can also act to confer insulin resistance in skeletal muscle. Therefore, the role of each arm of UPR needs to be further investigated to understand the molecular basis of cancer cachexia. It is noteworthy that most of the studies regarding the regulation of ER stress and the UPR in cancer cachexia are performed using cultured mammalian cells or preclinical animal models. While animal models and cell culture studies are important to understand the molecular mechanisms, additional studies in patients are required to have corroborative clinical data about ER stress, the UPR, and cancer cachexia.
